# Tailored Therapies for Cardiogenic Shock in Hypertrophic Cardiomyopathy: Navigating Emerging Strategies

**DOI:** 10.3390/jcdd11120401

**Published:** 2024-12-11

**Authors:** George E. Zakynthinos, Ioannis Gialamas, Vasiliki Tsolaki, Panteleimon Pantelidis, Athina Goliopoulou, Maria Ioanna Gounaridi, Ioanna Tzima, Andrew Xanthopoulos, Konstantinos Kalogeras, Gerasimos Siasos, Evangelos Oikonomou

**Affiliations:** 1 3rd Department of Cardiology, “Sotiria” Chest Diseases Hospital, Medical School, National and Kapodistrian University of Athens, 11527 Athens, Greece; jyialamas@gmail.com (I.G.); pan.g.pantelidis@gmail.com (P.P.); agoliopoulou@gmail.com (A.G.); mar.gounaridi@gmail.com (M.I.G.); ioannatzima92@gmail.com (I.T.); kalogerask@yahoo.gr (K.K.); ger_sias@hotmail.com (G.S.); boikono@gmail.com (E.O.); 2 Critical Care Department, University Hospital of Larissa, Faculty of Medicine, University of Thessaly, 41335 Larissa, Greece; vasotsolaki@yahoo.com; 3 Department of Cardiology, University Hospital of Larissa, Faculty of Medicine, University of Thessaly, 41110 Larissa, Greece; andrewvxanth@gmail.com; 4 Cardiovascular Division, Brigham and Women’s Hospital, Harvard Medical School, Boston, MA 02115, USA

**Keywords:** hypertrophic cardiomyopathy, cardiogenic shock, mechanical circulatory support, left ventricular outflow tract obstruction, beta-blockers, extracorporeal membrane oxygenation

## Abstract

Hypertrophic cardiomyopathy (HCM) is a complex and heterogeneous cardiac disorder, often complicated by cardiogenic shock, a life-threatening condition marked by severe cardiac output failure. Managing cardiogenic shock in HCM patients presents unique challenges due to the distinct pathophysiology of the disease, which includes dynamic left ventricular outflow tract obstruction, diastolic dysfunction, and myocardial ischemia. This review discusses current and emerging therapeutic strategies tailored to address the complexities of HCM-associated cardiogenic shock and other diseases with similar pathophysiology that provoke left ventricular outflow tract obstruction. We explore the role of pharmacological interventions, including the use of vasopressors and inotropes, which are crucial in stabilizing hemodynamics but require careful selection to avoid exacerbating the outflow obstruction. Additionally, the review highlights advancements in mechanical circulatory support devices such as extracorporeal membrane oxygenation (ECMO) and left ventricular assist devices (LVADs), which have become vital in the acute management of cardiogenic shock. These devices provide temporary support and bridge patients to recovery, definitive therapy, or heart transplantation, which remains a critical option for those with end-stage disease. Furthermore, the review delves into the latest research and clinical trials that are refining these therapeutic approaches, ensuring they are optimized for HCM patients. The impact of these treatments on patient outcomes, including survival rates and quality of life, is also critically assessed. In conclusion, this review underscores the importance of a tailored therapeutic approach in managing cardiogenic shock in HCM patients, integrating pharmacological and mechanical support strategies to improve outcomes in this high-risk population.

## 1. Introduction

Hypertrophic cardiomyopathy (HCM) is an inherited cardiovascular disorder with highly variable clinical presentations. The Coronary Artery Risk Development in Young Adults (CARDIA) cohort study reported a prevalence of approximately 1 in 500 individuals [1], with newer research suggesting that this figure may be even higher [2]. When clinical and genetic diagnostic tools are combined, the prevalence of HCM is estimated to reach as high as 1 in 200 people [3]. Globally, around 20 million individuals, including roughly 750,000 in the United States, are believed to be living with HCM. This condition is up to 35 times more common than other inherited cardiovascular diseases such as arrhythmogenic right ventricular cardiomyopathy, Marfan syndrome, dilated cardiomyopathy, and ion channel disorders [4]. These statistics underscore the significant impact of HCM.

Despite substantial advances in the diagnosis and treatment of HCM, patients still face a higher risk of mortality compared to the general population. A large European cohort study found that individuals with HCM have a standardized mortality ratio of 2.0, meaning they are twice as likely to die as age- and sex-matched counterparts [5]. Although treatments such as implantable cardioverter-defibrillators (ICDs) and septal reduction procedures have lowered rates of sudden cardiac death (SCD) and improved patient outcomes, mortality remains elevated, particularly among women [5]. While sudden cardiac death accounts for the majority of HCM-related deaths, nearly 25% of patients die from heart failure [6].

Beyond its clinical impact, HCM also imposes an economic burden on healthcare systems. A US study assessing the financial costs associated with HCM revealed an average annual healthcare cost of USD 8500 per patient, significantly higher than that of the general population [7]. Inpatient hospitalizations comprise 54% of total costs, while emergency room visits account for approximately 13%, and outpatient visits contribute 33% [7]. Frequent hospitalizations, diagnostic imaging, and interventions such as ICDs and septal reduction procedures drive these high costs. Furthermore, about 60% of HCM patients require ongoing, long-term monitoring, adding to the strain on healthcare resources [7]. This highlights the need for more efficient management strategies to reduce the economic and clinical burden of HCM.

Historically, HCM was viewed as a severe and often lethal disease with a few effective treatment options. However, in the last two decades, the clinical understanding of HCM has transformed significantly. Advances in early recognition, including the identification of low-risk subgroups with minimal symptoms or disability, alongside improved treatment strategies, have drastically changed patient outcomes [3,8]. Mortality and morbidity have declined, with many patients now able to live into their 70s, 80s, and even 90s with a good quality of life [8,9,10,11,12]. Personalized therapies targeting specific disease mechanisms have helped reduce HCM-related mortality by more than tenfold, from an early rate of 6% per year to just 0.5% annually [10,13,14]. This mortality rate is now among the lowest for major diseases, comparable to those of cancer, neurological disorders, and heart failure. With the range of modern management strategies available, death directly attributable to HCM has become rare, primarily affecting a small subset of patients with non-obstructive forms of the disease who suffer from advanced, treatment-resistant heart failure [15].

However, despite the importance of HCM, its elevated prevalence, and the skyrocketing economic burden it imposes on healthcare systems, there is still limited research and insufficient guidelines addressing the management of cardiogenic shock in HCM patients. Indeed, despite these advancements in managing HCM, there remains a notable gap in data regarding the management of these critical areas. A few studies address the use of circulatory support devices in these cases, and both European and American guidelines lack detailed recommendations for treating cardiogenic shock in HCM. The purpose of this review is to illuminate this underexplored field and provide much-needed insights into the management of cardiogenic shock in HCM patients, offering a framework for better understanding and clinical decision-making in this complex condition.

## 2. Pathophysiology

Pathophysiology of Hypertrophic Cardiomyopathy:

Hypertrophic cardiomyopathy in adults is characterized by a left ventricular (LV) wall thickness exceeding 15 mm in any myocardial segment, which cannot be solely attributed to loading conditions such as hypertension or valvular disease. In some cases, wall thicknesses of 13–14 mm may also indicate HCM, necessitating further assessment that includes family history, genetic testing, and electrocardiogram (ECG) findings [16,17].

Many individuals with HCM inherit the condition through an autosomal dominant pattern, typically due to mutations in genes encoding various cardiac sarcomere proteins. These mutations often result in an asymmetrical hypertrophy pattern, primarily affecting the interventricular septum and leading to disorganization of myocytes. However, this thickening is not solely due to cardiomyocyte hypertrophy but also involves an increase in extracellular matrix. This disarray can impair electrical conduction and elevate the risk of arrhythmias [18,19]. Additionally, the dimensions of the left ventricular cavity are often diminished, while fractional shortening may be increased due to the thickened myocardium. This results in reduced ventricular filling and elevated diastolic pressures, culminating in diastolic dysfunction [18,19].

Asymmetric thickening of the hypertrophied septum may obstruct blood flow from the LV to the aorta, causing dynamic left ventricular outflow tract (LVOT) obstruction, which may also be mid-cavitary. During periods of increased physiological demand, such as exercise, this obstruction can exacerbate hemodynamic compromise and provoke symptoms like dyspnea or syncope [16,19]. Furthermore, patients with HCM often exhibit systolic anterior motion (SAM) of the mitral valve, primarily induced by the left ventricular flow impacting the protruding mitral valve leaflet, which further contributes to the outflow obstruction [20,21,22].

Chronic myocardial stress in patients with HCM leads to interstitial fibrosis, which impairs ventricular relaxation and worsens diastolic dysfunction, significantly in hypertrophy, and fibrosis also compromises coronary blood flow reserve, elevating the risk of ischemia even in the absence of significant coronary artery disease. A small percentage of patients (up to 10% in certain studies) may progress to left ventricular enlargement and systolic dysfunction [17].

**Figure 1:**
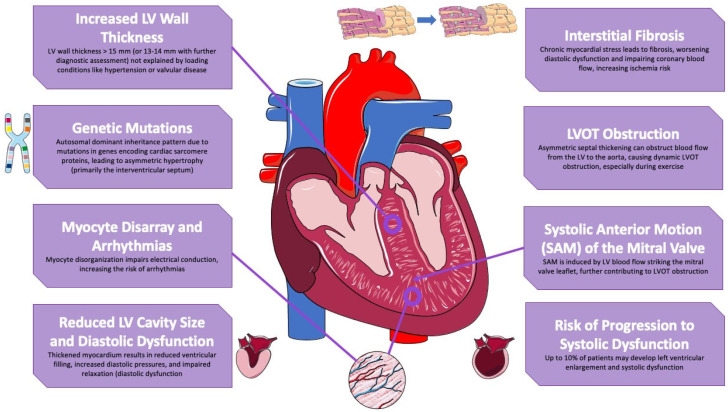
The figure illustrates the key physiological changes in hypertrophic cardiomyopathy (HCM). It highlights increased left ventricular (LV) wall thickness, often due to genetic mutations affecting sarcomere proteins. The resulting myocyte disarray contributes to arrhythmias, while thickened myocardium leads to reduced LV cavity size and diastolic dysfunction. Dynamic LV outflow tract (LVOT) obstruction and systolic anterior motion (SAM) of the mitral valve further impair hemodynamics. Chronic stress promotes interstitial fibrosis, exacerbating diastolic dysfunction, with some patients progressing to systolic dysfunction. Parts of the figure were drawn by using pictures from Servier Medical Art. Servier Medical Art by Servier is licensed under a Creative Commons Attribution 4.0 Unported License (https://creativecommons.org/licenses/by/4.0/, accessed on 9 October 2024).

Patients with HCM often exhibit a unique phenomenon known as “paradoxical” autonomic dysfunction, which plays a critical role in the pathophysiology of the disease. This dysfunction can lead to inappropriate vasodilation and a subsequent drop in blood pressure (BP), which are counterproductive in maintaining adequate hemodynamics. In the context of HCM, this vasodilatory response exacerbates the obstruction of the left ventricular outflow tract (LVOTO) by further reducing the preload and afterload, which in turn increases the dynamic nature of the obstruction [17]. The interplay between autonomic imbalance and LVOTO underscores the complex mechanisms that contribute to symptom exacerbation, particularly during states of stress, exercise, or dehydration, where these effects are magnified. Recognizing and addressing this autonomic dysregulation is essential in the comprehensive management of patients with HCM [18] (Figure 1).

Pathophysiology of Cardiogenic Shock in Hypertrophic Cardiomyopathy:

The definitions of CS have evolved significantly over time, particularly emphasized by the SHOCK Trial (1999) [23] and the FRENSHOCK trial (2022) [24]. The SHOCK Trial established a definition based on clinical criteria of systolic blood pressure (SBP) <90 mmHg and end-organ hypoperfusion, alongside hemodynamic criteria of cardiac index (CI) of <2.2 L/min/m^2^ and pulmonary capillary wedge pressure (PCWP) of ≥15 mmHg [23]. This approach focused on both systemic effects and physiological parameters. The IABP-SHOCK II trial (2012) expanded the definition to include catecholamine use and clinical pulmonary congestion, reflecting a broader understanding of the condition [25]. The CARDSHOCK trial (2015) emphasized acute cardiac causes and specific signs of hypoperfusion, such as elevated lactate levels [26]. In contrast, the FRENSHOCK trial refined the definition to incorporate SBP of <90 mmHg or CI of <2.0 L/min/m^2^, along with elevated heart pressures and clear signs of hypoperfusion [24].

These evolving definitions have paved the way for the development of the Society for Cardiovascular Angiography and Interventions (SCAI) 5-stage classification of shock [27]. This framework categorizes shock into five distinct stages—ranging from stage A (pre-shock) to stage E (end-stage shock)—providing a structured approach to assess the severity and management of patients with cardiogenic shock [27]. This classification aims to enhance communication among healthcare providers and guide therapeutic interventions, ultimately improving patient outcomes in this critical condition.

The pathophysiology of CS in HCM does not conform entirely to the classic clinical features of CS and can be divided into two distinct entities. The first group includes patients without obstructive physiology, who may finally develop systolic dysfunction. In these cases, the underlying pathophysiology aligns with that of typical CS, allowing for the application of established treatment and classification protocols, such as the use of inotropes, vasoconstrictors, and circulatory support devices [16]. However, this form of CS is relatively uncommon in HCM. The predominant entity, by contrast, is CS due to LVOT obstruction (LVOTO), which represents a fundamentally different pathophysiological process.

Cardiogenic shock due to LVOTO may be easily provoked. In situations where preload falls, such as hypovolemia after exercise due to sweating or hemorrhage, obstruction can become profound. Even in cases of mild hypovolemia, this condition may be triggered [9]. Additionally, LVOTO can be exacerbated by inappropriately decreasing afterload, for example, through the use of afterload-reducing medications [9]. These hemodynamic changes further accentuate the dynamic nature of the obstruction, potentially leading to severe clinical deterioration.

Obstructive hypertrophic cardiomyopathy (HOCM) is defined by a peak instantaneous Doppler LVOT gradient of 30 mmHg or higher. However, theoretical models suggest that this gradient becomes hemodynamically significant when it reaches or exceeds 50 mmHg [28,29], at which point invasive treatment for the obstruction is often recommended. Obstruction of the LVOT primarily results from the combination of abnormal interventricular septal thickening and the anatomical positioning of the mitral valve [3,30]. The prominent hypertrophy reduces the outflow pathway, creating a narrowed channel through which blood must flow during systole. As blood is ejected from the left ventricle, it encounters the anteriorly displaced mitral valve leaflets, which can protrude into the outflow tract. This anatomical setup predisposes the mitral valve to experience significant hydrodynamic forces [31].

Contrary to the traditional Venturi hypothesis, recent studies indicate that SAM of the mitral valve begins at relatively low velocities in the LVOT, around 89 cm/s, which is comparable to normal patients without SAM [20]. This challenges the assumption that high-velocity flow is necessary to create the under-pressure that supposedly pulls the mitral valve leaflet towards the septum. Instead, the data suggest that drag forces—rather than lift forces associated with Venturi effects—are more critical in the development of SAM [20]. As blood flows through the narrowed LVOT, drag forces, which act in the direction of the flow, exert a pushing effect on the mitral valve, contributing to its motion towards the septum [20].

The geometry of the mitral valve in HCM, particularly its anterior positioning and larger size, further amplifies the impact of these drag forces. The protruding leaflets effectively intercept the high-velocity flow, creating a significant interaction with the ejection flow [31]. This leads to a dynamic situation where the mitral valve is pushed towards the septum during systole, exacerbating the LVOTO [20]. Additionally, this positioning often results in mitral regurgitation due to the valve’s altered position; however, this regurgitation is typically mid- to end-systolic and does not contribute significantly to the hemodynamic burden. As the obstruction intensifies, the heart must work harder to eject blood, leading to increased left ventricular pressures and decreased cardiac output. If this condition worsens, it can culminate in cardiogenic shock, characterized by the heart’s inability to maintain adequate perfusion to meet the body’s demands [32]. The resultant low cardiac output can cause significant clinical symptoms, such as dyspnea and fatigue, and, in severe cases, may result in life-threatening hemodynamic instability [32]. Dyspnea occurs primarily due to lung congestion caused by elevated left atrial pressure and pulmonary venous hypertension and indirectly due to reduced cardiac output, which further impairs oxygen delivery to tissues.

In HCM, severe obstruction can precipitate episodes of LV apical ballooning, a phenomenon observed in some patients with dynamic outflow obstruction [33]. This condition is characterized by a sudden dilation of the LV apex, leading to a presentation that mimics acute coronary syndrome, including symptoms such as chest pain, shortness of breath, and syncope [33]. In a study of 13 patients with HCM and dynamic obstruction, episodes of apical ballooning were associated with high LVOT gradients averaging 92 ± 37 mm Hg [34]. This acute event typically occurs in the context of exertion or physiological provocation, such as the Valsalva maneuver, where decreased preload and increased afterload exacerbate the obstruction and trigger severe systolic dysfunction [34]. The physiology behind apical ballooning in HCM involves a complex interplay of high LVOT gradients and dynamic changes in myocardial function [35]. When the LVOT becomes significantly obstructed, especially during periods of increased demand, the heart struggles to eject blood effectively, leading to a marked reduction in forward flow and subsequent ischemia [33,34]. This drop in ejection velocity can culminate in apical dilation and hypokinesia, as the apex becomes a site of stasis, trapping blood and resulting in an abnormal motion pattern. Echocardiographic findings often reveal mid-LV akinesia or hypokinesia, with preserved or even enhanced contractility in the basal segments [33,34]. Thus, the acute onset of LV apical ballooning highlights the urgent need for effective management strategies to relieve obstruction, as surgical intervention has been shown to restore normal function and reverse the ballooning phenomenon.

Understanding the mechanisms behind apical ballooning is critical, as timely recognition and treatment of LVOTO can prevent progression to severe cardiogenic shock or refractory heart failure.

As previously mentioned, obstruction in HCM can lead to ischemia, ultimately resulting in apical ballooning. It was traditionally believed that ischemia stemmed from several potential mechanisms, including increased oxygen demands due to myocardial hypertrophy, impaired ventricular relaxation, anatomical abnormalities of the intramyocardial arterioles, and LVOTO [36,37,38]. However, recent data indicate that the relationship between obstruction and ischemia may be more nuanced.

Microvascular ischemia in HCM is linked to a combination of mechanisms that exacerbate myocardial oxygen supply-demand mismatches [39]. First, increased wall thickness from hypertrophy leads to elevated myocardial oxygen requirements while simultaneously compressing intramyocardial microcirculation during systole. This compression results in a significant backward compressive wave (BCW) (Table 1) that can disrupt coronary flow [39]. Second, impaired ventricular relaxation decreases the effective driving pressure during diastole, contributing to diminished coronary perfusion. Third, transient LVOTO creates fluctuations in proximal driving pressures, further compromising forward flow in the coronary arteries [39].

Additionally, the role of an intramural deep course of the descending coronary artery further complicates myocardial perfusion. This anatomical variation can predispose to localized ischemia by restricting effective coronary blood flow [37]. Functional small vessel obstruction during diastole, driven by diastolic restriction and elevated ventricular pressures, also plays a significant role in limiting perfusion. Histological abnormalities in the microcirculatory vessels have been observed, indicating that structural changes further contribute to ischemia [38].

The cumulative effect of these factors—impaired relaxation, high intracavitary pressures, abnormal microvascular structure, and functional vessel obstruction—leads to significant myocardial perfusion abnormalities [39].

The dynamics of coronary blood flow are significantly altered due to the condition’s unique pathophysiological features. During systole, the marked myocardial hypertrophy and elevated intracavitary pressures compress the intramyocardial vasculature, potentially causing a reversal of coronary flow [37]. This phenomenon reflects the substantial mechanical forces that oppose forward blood flow within the coronary arteries during contraction. Conversely, diastole becomes the critical phase for myocardial perfusion in HCM, with coronary blood flow substantially increasing to compensate for the systolic deficit [36,37]. The exaggerated reliance on diastolic perfusion underscores the vulnerability of the hypertrophied myocardium to ischemia, particularly when diastolic filling time is shortened, such as in tachycardia [38]. Understanding these unique flow characteristics is essential for optimizing therapeutic strategies aimed at preserving coronary perfusion and mitigating ischemic risks in patients with HCM.

## 3. Diagnosis of Cardiogenic Shock

Diagnosis of cardiogenic shock in HCM relies upon the same principles as in every other condition, such as signs of low cardiac output, such as hypotension (systolic blood pressure <90 mmHg), cold and clammy extremities, altered mental status, oliguria, and increased jugular venous pressure and laboratory findings such as metabolic acidosis and elevated lactate levels, reflecting poor tissue perfusion and anaerobic metabolism [40,41]. Serum biomarkers of myocardial injury, such as elevated troponins, are common and indicate myocardial ischemia or infarction as a potential cause. Additionally, B-type natriuretic peptide (BNP) or N-terminal pro-BNP (NT-proBNP) levels are typically elevated, reflecting increased ventricular wall stress [40,41].

However, as previously mentioned, it is essential to identify cardiogenic shock due to LVOT obstruction in patients with HCM. To diagnose these patients, specific echocardiographic findings may be utilized, such as continuous-wave Doppler to measure peak LVOT gradients, with a reading of ≥30 mmHg at rest or following provocation indicating significant obstruction [42,43]. In our case, cardiogenic shock itself acts as the provocateur. The Doppler waveform typically exhibits a dagger shape, reflecting the high-velocity flow and timing of mitral-septal contact [35,42]. Additionally, SAM of the mitral valve leaflets should be evaluated, as this motion often contributes to LVOTO [43]. The presence of a “lobster claw sign” can also be noted, signifying a mid-systolic drop in left ventricular ejection velocities due to significant outflow obstruction [44]. Other findings may include the development of apical aneurysms, which can manifest as akinetic or dyskinetic segments in severe cases [43].

Cardiac magnetic resonance imaging (MRI) plays a pivotal role in diagnosing HCM, offering unparalleled insights into myocardial structure and function [43]. MRI is particularly valuable for detecting myocardial fibrosis through late gadolinium enhancement and for identifying subtle hypertrophic patterns, such as apical or focal hypertrophy, that may be missed on echocardiography [43]. However, due to the logistical challenges and hemodynamic instability often present in cardiogenic shock, the use of MRI in such scenarios is limited. Therefore, its role will not be further analyzed in this review.

## 4. Treatment

The first step, in order to treat patients with HCM and LVOTO, is to give a high amount of fluids to increase preload, in which most patients are sensitive and symptoms decline [3,15]. By administering IV crystalloids, preload is increased, expanding the left ventricular cavity and helping to reduce the degree of LVOT obstruction. This preload augmentation can stabilize hemodynamics by reducing the left ventricular wall contact with the septum during systole, thereby diminishing the SAM of the mitral valve. SAM, often worsened in low preload states, leads to worsened obstruction and potentially severe mitral regurgitation, compounding the hemodynamic burden in these patients [16,19].

Raising preload also influences afterload, which becomes crucial in cardiogenic shock. An elevated afterload counteracts the hyperdynamic ejection in HCM patients, allowing the left ventricle to contract against a steadier resistance [34]. This reduces the outflow tract gradient, alleviates obstruction, and enhances overall cardiac output. The combined effect of increased preload and modestly elevated afterload can markedly improve perfusion pressure and mitigate the symptoms of cardiogenic shock, stabilizing blood pressure and supporting vital organ perfusion [34].

In addition, it is very important to manage new or poorly treated atrial fibrillation (AF), which can aggravate LVOTO-related symptoms, so it is important to swiftly restore sinus rhythm or control the heart rate. The treatment of AF in cardiogenic shock follows standard protocols, with electrical cardioversion as the first choice due to instability. If cardioversion is not feasible, beta-blockers and other antiarrhythmics, such as amiodarone, are considered. Additionally, drugs like digoxin should be avoided due to their potential to increase the heart’s contractility, which can worsen LVOTO [9]. However, in this review, we will not analyze AF management, because it is out of the scope of the text. If fluids fail, pharmaceutical treatment is the next step.

Our knowledge in the treatment of cardiogenic shock due to HCM is limited due to its complexity and the small number of cases. As a result, our understanding also draws from other conditions where LVOT obstruction may be significant, such as Takotsubo cardiomyopathy or myocardial infarction with LVOT obstruction. This review will analyze all these conditions.

### 4.1. Pharmaceutical Treatment

#### 4.1.1. Beta-Blockers

Beta-blockers have been a cornerstone in managing symptoms in patients with HCM for decades. Initially, propranolol, a non-selective beta-blocker, demonstrated its efficacy in alleviating symptoms in HCM patients, even though it did not modify the disease progression [45]. Over time, more selective beta-blockers like metoprolol have become first-line treatments. In managing LVOTO, non-vasodilating beta-blockers are preferred, as they mitigate symptoms by decreasing heart rate and reducing left ventricular contractility [16]. While beta-blockers are widely prescribed, a few studies compare their specific effects directly. However, metoprolol has gained recognition due to its ability to reduce LVOT gradients both at rest and during exertion, LVOT gradient metoprolol vs. placebo at rest [46].

A notable double-blind, placebo-controlled study evaluated the effects of metoprolol in 29 patients with obstructive HCM. The results showed that metoprolol significantly reduced LVOT gradients at rest (25 mm Hg vs. 72 mm Hg, *p* < 0.007) and during peak exercise (28 mm Hg vs. 62 mm Hg, *p* < 0.001), providing symptomatic relief and improving patients’ quality of life. Importantly, the study highlighted its effectiveness even in higher oxygen demand situations like exercise [46], suggesting potential use in high-stress conditions such as shock. This reduction in gradients is a key finding, as LVOTO has been closely linked to the severity of symptoms, including exertional dyspnea and chest pain. Metoprolol also improved left ventricular longitudinal strain (LVLS), particularly in the apical and mid-ventricular segments, showing that β-blocker therapy positively impacts left ventricular function during exercise [47]. Additionally, metoprolol was shown to reduce heart rate (*p* < 0.0001), mitigate mitral regurgitation (*p* = 0.004), and decrease the LVOT gradient (*p* = 0.0005) during exercise while increasing stroke volume (+9 mL; 95% CI: 2–15 mL; *p* = 0.008) [48]. These findings suggest that metoprolol, and possibly other beta-blockers, may be beneficial not only during exercise-induced stress but also in other high-stress conditions such as cardiogenic shock, further supporting its broader application in managing hemodynamic challenges.

Carvedilol, though less commonly used in HCM, offers paradoxically a unique advantage due to its dual β- and α1-adrenergic blocking properties. A recent study identified that R-carvedilol, by inhibiting RyR2 (ryanodine receptor type 2), effectively reduces hyperdynamic contraction, arrhythmias, and LV contractility while maintaining stroke volume [49]. Its R-enantiomer, which lacks β-blocking properties, is particularly beneficial, as it can suppress hyperdynamic contractions and arrhythmias without lowering heart rate. In mouse models of HCM, R-carvedilol was superior to metoprolol, verapamil, and mavacamten in normalizing cardiac output and preventing fatal arrhythmias. This makes carvedilol, particularly its R-enantiomer, an intriguing therapeutic option for HCM patients, especially those with non-obstructive HCM, where traditional treatment focuses primarily on managing symptoms of obstruction [49].

Even in low blood pressure cases, β-blockers, including intravenous metoprolol, are still employed. Contrary to initial concerns, using metoprolol in patients with systolic pressures as low as 80 mmHg has shown that LV outflow gradients often decrease, and blood pressure either increases or remains stable. In fact, a series of 7 patients demonstrated this effect, where intravenous metoprolol (15 mg over 15 min) safely reduced obstruction while keeping hemodynamic stability intact [50]. For patients whose systolic pressures fall below 80 mmHg, combining phenylephrine with metoprolol ensures the continued benefits of β-blockade while providing blood pressure support [50]. This highlights the flexibility of β-blocker therapy in managing outflow obstructions, even under challenging hemodynamic conditions. It is important to note that when β-blockers are used in patients with SBP in the 1980s, phenylephrine should be readily available for concurrent administration to maintain hemodynamic stability.

For patients experiencing acute hemodynamic instability, short-acting beta-blockers such as esmolol (half-life: 9 min) and landiolol (half-life: 4 min) have been employed with significant success, due also to their rapid effect. Esmolol, due to its short half-life, is frequently used in perioperative settings or during acute cardiac events, providing rapid control over heart rate and contractility. Case reports highlight its use during high-risk procedures, such as labor and cardiopulmonary bypass in HCM patients, where it stabilized blood pressure and helped manage left ventricular outflow tract obstruction (Table 2) [51,52,53].

Landiolol, a more selective β1-blocker with a short duration of action, has shown promise in similar scenarios. In cases of Takotsubo cardiomyopathy and HCM-related complications during cesarean sections, landiolol infusion quickly stabilized heart rate, reduced LVOT gradients, and improved blood pressure, demonstrating its utility in managing acute episodes in patients with HCM and left ventricular outflow obstruction [54,55]. In one case involving a cesarean section, landiolol infusion resulted in a gradual decrease in heart rate and an increase in blood pressure within just five minutes, highlighting its rapid efficacy [56]. Furthermore, landiolol was effective in cases of Takotsubo cardiomyopathy, where LVOTO and mitral regurgitation resulted in significant hemodynamic instability. By reducing the LVOT gradient, landiolol helped restore hemodynamic stability in these patients [55].

In summary, beta-blockers, ranging from long-standing agents like propranolol to newer, more selective options such as landiolol and carvedilol, remain central to managing hypertrophic cardiomyopathy (HCM). This may be explained by wave intensity analysis (WIA), which examines the waves responsible for the acceleration or deceleration of coronary blood flow [57]. WIA may clarify the improvement in myocardial perfusion observed with beta-blockers and calcium channel blockers, as both agents increase diastolic duration and reduce myocardial contractility, theoretically leading to a reduction in backward compression waves (BCWs) [58]. Even in cases where cardiogenic shock is prominent, beta-blockers can remain a first-line option. Additionally, short-acting beta-blockers, such as esmolol and landiolol, provide valuable therapeutic options in acute settings, particularly in managing perioperative risks or sudden cardiac decompensation.

**Table 2:** The table summarizes various cases and series of patients with hypertrophic cardiomyopathy (HCM), left ventricular outflow tract obstruction (LVOTO), and related conditions, highlighting the use of beta-blockers as a therapeutic option in patients experiencing cardiogenic shock and hypotension.

Data on Patients with HCM					
**Case/Series**	**Number of** **Cases**	**Description**	**Beta-Blocker Used**	**Outcome**	**Blood Pressure (BP)** **(Pre-Post)**
Sherrid MV. et al. [50], 2021—**HOCM** with apical ballooning	7	- Mainstay therapy for apical ballooning - Intravenous beta-blocker therapy - Effective in borderline or low blood pressure	Metoprolol	LVOT gradients often decreased, and blood pressure remained unchanged	<90 mmHg–stable or increase in BP
Tsukano Y. et al. [54], 2015—**HOCM** during cesarean section	1	- Development of dyspnea, tachycardia, and hypotension after uterotonic drug - Improvement with landiolol infusion	Landiolol	Improved symptoms and hemodynamic stabilization	Hypotension–blood pressure stabilized
Abe T. et al. [56], 2010—**HOCM** post-cesarean section	1	- Tachycardia after delivery - Blood pressure increases within 5 min	Landiolol	Blood pressure stabilized, and heart rate reduced.	90/45 mmHg–120/60 mmHg
Fairley CJ. et al. [51], 1995—Severe **HOCM** during vaginal delivery	1	- Esmolol use during labor in HCM patient - Assisted vaginal delivery with minimal disturbance	Esmolol	Successful delivery of fetus Both mother and fetus were safe	β-blocker started when “reduction in systemic arterial pressure was observed”
Ooi LG. et al. [52], 1993—**HOCM** post-cardiopulmonary bypass	1	- Hypotension during weaning from bypass in HCM patient - Administration of esmolol - Successful second attempt to wean off bypass	Esmolol	Successful weaning of cardiopulmonary bypass Patient extubated 24 h later Continued with propanol	80/60 mmHg–130/80 mmHg
**Data on patients with LVOTO, without HCM**					
Chen C. et al. [53], 2024—LVOTO misdiagnosed as ACS	1	- Symptoms: chest pain, syncope, and shock - ST depression in leads V5–V6, II, and aVF, and ST elevation occurred in lead aVR - Symptoms relieved with esmolol	Esmolol	Blood pressure normalized within 30 min of treatment The patient rejected septal myectomy or alcohol ablation and received endocardial radiofrequency ablation of septal hypertrophy	Hemodynamically unstable before esmolol–stable 30 min after infusion started
Cho Y. et al. [55], 2023—Takotsubo Cardiomyopathy with LVOTO	3	- LVOT obstruction with shock - Landiolol infusion effective gradient release - Hemodynamic stability improvement	Landiolol	Successful reduction in LVOT gradient, leading to improved hemodynamics	90.3 ± 18.6 mmHg–100.7 ± 16.8 mmHg
Rafiq I. et al. [59], 2020—LVOTO post-Ebstein surgery	1	- Patient hypotensive, oliguric, and acidotic, despite adequate fluid resuscitation, after surgical repair of Ebstein - LVOT observed via emergency TEE - Significant SAM with severe mitral regurgitation	Esmolol	Significant improvement in LVOT obstruction and hemodynamic status Hemodynamics improved within minutes of starting infusion	<70 mmHg–>100 mmHg
Santoro F. et al. [60], 2016—LVOTO in patients with Takotsubo Cardiomyopathy	10 (LVOT obstruction) 1 Hypotension	- LVEF at admission 35 ± 9%, which significantly improved at discharge (53 ± 4%, *p* = 0.001). - LVOT pressure gradient before esmolol: 47.6 ± 16.6 mmHg, after: 18.2 ± 2.3 mmHg (*p* = 0.0091)	Esmolol	Improvements in LV function, LVOT gradient, and hemodynamic status	1 patient: 80/50 mmHg–not mentioned All other patients with systolic BP > 90 mmHg.
Angue M. et al. [61], 2014—Takotsubo syndrome with LVOTO	1	- 71-year-old woman with chest pain, severe MR, and apical ballooning - Treatment included dobutamine, IABP, esmolol, and intubation - Basal hyperkinesis and significant septal bulge-induced LVOTO and severe MR on a SAM of the mitral anterior leaflet	Esmolol	Good clinical outcome, extubated three days later successful weaning from inotropes with the administration of esmolol Improvement in cardiac index and clinical outcome after esmolol administration Extubated three days later with per os metoprolol	80/45 mmHg–not mentioned
Migliore F. et al. [62], 2010—Takotsubo syndrome with dynamic LVOTO	1	- Patient with apical and mid-wall left ventricular akinesis, basal hyperkinesis, moderate systolic dysfunction, and severe MR - SAM of the mitral anterior leaflet - Cardiogenic shock	Metoprolol	Improvement in LVOTO and overall cardiac function One day after discharge, the patient is asymptomatic and doing well Resolution of the regional systolic dysfunction with a normal left ventricular ejection fraction	Patient in cardiogenic shock (BP not mentioned)
Fefer P. et al. [63], 2009—Takotsubo cardiomyopathy and LVOTO	1	- ST-segment elevation in the anterior leads, but no evidence of obstructive coronary disease - Pullback of the catheter across the LVOT and aortic valve demonstrated gradient 70 mmHg - Contrast ventriculogram showed apical ballooning with hyperdynamic function of the basal constrictors	Metoprolol	Intravenous administration of 5 mg metoprolol resulted in a rise in systolic blood pressure to approximately 100 mmHg and reduced the outflow tract gradient to around 50 mmHg Oral metoprolol was initiated Repeat echocardiogram 3 days later showed apical hypokinesis but essentially no outflow tract gradient	Systolic BP: 75 mmHg–100 mmHg
Taylor JS. [64], 2009—Acquired LVOT obstruction in ACS (not HCM)	1	- Acute coronary syndrome with critical cardiogenic shock and LVOTO - Treated with intravenous beta-blockers	Esmolol	Clinical and hemodynamic improvement, resolution of LVOT obstruction Discharged 10 days later	62/34 mmHg–>120/60 mmHg
Kushikata T. et al. [65], 2006—LVOT obstruction post-mitral valve plasty	1	- Severe cardiovascular collapse after mitral valve plasty due to LVOTO - Treated with volume loading and landiolol	Landiolol	Successful treatment of LVOT obstruction with improved hemodynamics Landiolol was effective for nonsurgical treatment of LVOT obstruction due to SAM.	
**Data on patients with HCM but without LVOTO**					
Yoshioka T. et al. [66], 2008—Dynamic midventricular obstruction (MVO) * in Takotsubo patients	34	- Persistent hypotension with MVO in transient LV apical ballooning - Propranolol challenge effective in reducing MVO	Propranolol	Significant reduction in pressure gradient and improvement in systolic blood pressure and LVEF	Systolic BP: 85 ± 11 mmHg–116 ± 20 mmHg

Abbreviations: HCM—hypertrophic cardiomyopathy; LVOTO—left ventricular outflow tract obstruction; BP—blood pressure; LVEF—left ventricular ejection fraction; SAM—systolic anterior motion; IABP—intra-aortic balloon pump; ACS—acute coronary syndrome. * MVO is mentioned due to similar pathophysiology.

#### 4.1.2. Calcium Channel Blockers

Calcium channel blockers (CCBs) inhibit L-type calcium channels in cardiomyocytes and vascular smooth muscle, promoting vasodilation and reducing inotropic and chronotropic activity [67]. This reduction in calcium influx into ventricular myocytes decreases contractility, while slowing sinoatrial node firing reduces heart rate. They have been generally used in patients with LVOTO in steady state; however, data on their use in cardiogenic shock do not exist. Verapamil and diltiazem also slow atrioventricular conduction by prolonging the refractory period in the AV node. However, these medications must be used cautiously in patients with hypotension, AV block, or atrial fibrillation, as they can aggravate heart failure or conduction disturbances in predisposed patients [67].

Non-dihydropyridine CCBs, particularly verapamil and diltiazem, have been used since the 1970s to manage HCM, especially in cases resistant to beta-blockers [68,69]. Both agents improve symptoms, with verapamil increasing exercise capacity by 26% [70]. However, in patients with significant left ventricular outflow tract obstruction, verapamil may increase the risk of sudden cardiac death (SCD), as shown by Epstein et al. [71]. Thus, while CCBs can be beneficial in managing HCM, their use in acute settings like cardiogenic shock has not been tested, necessitating further randomized controlled trials to evaluate their safety and efficacy in such emergencies.

#### 4.1.3. Disopyramide

Disopyramide is an effective antiarrhythmic drug used in the management of HCM, particularly for reducing LVOTO [72]. It has been shown to reduce symptoms and is associated with a low risk of SCD [73]. Additionally, it has been safely used in pediatric patients with LVOTO, further supporting its versatility in treating HCM [74]. However, disopyramide lacks an intravenous (IV) formulation, limiting its use in acute emergency settings such as cardiogenic shock. Due to the absence of an IV version, disopyramide is not commonly utilized in critical care or emergency situations, and there is limited clinical data or publications supporting its role in these settings.

Nevertheless, Shirak et al. described a case where oral disopyramide was effectively used in a patient with obstructive HCM who had a history of COPD, which precluded the use of beta-blockers. This approach resulted in significant symptom relief and a marked reduction in the LVOT gradient from 92 mmHg to 25 mmHg within two hours [75]. This case underscores the potential role of oral disopyramide in managing acute exacerbations of obstructive HCM when beta-blockers are contraindicated.

#### 4.1.4. Inotropes

The use of inotropes in the management of HCM requires careful consideration due to the unique hemodynamic challenges associated with the condition. Dobutamine, while often utilized to enhance cardiac output, can inadvertently induce LVOTO, even in patients without HCM. Recent evidence indicates that LVOTO can occur due to factors such as severe hypovolemia or hyperkinesia, leading to changes in left ventricular shape, decreased preload, and increased heart rate during dobutamine infusion [76]. These changes can result in significant hemodynamic compromise.

Calcium sensitizers like levosimendan and pimobendan present additional complexities in HCM management. HCM is characterized by hypertrophy and increased myocardial contractility, which are exacerbated by enhanced calcium sensitivity at the myofilament level. This heightened sensitivity can lead to altered calcium fluxes and potential calcium trapping, which are implicated in arrhythmogenesis. Recent animal studies have demonstrated a direct correlation between increased calcium sensitivity and the risk of ventricular arrhythmias, particularly in transgenic models with troponin T mutations [77]. While these calcium sensitizers could theoretically provide short-term inotropic support, their potential to provoke arrhythmias in patients with HCM raises concerns about their safety in acute settings like cardiogenic shock. Therefore, the cautious use of inotropes, particularly those affecting calcium sensitivity, is critical in this patient population. Milrinone, while effective in improving cardiac output through its inotropic and vasodilatory effects, should be considered alongside these risks, emphasizing the need for individualized treatment strategies in HCM management. Due to these factors, inotropes should be avoided in HCM with LVOTO to prevent further hemodynamic compromise and arrhythmogenic events.

#### 4.1.5. Vasoconstrictors

In individuals with hypertrophic cardiomyopathy (HCM), β-adrenergic agonism can have a negative impact, as it may exacerbate symptoms by increasing heart rate and contractility, which in turn can worsen LVOTO and induce more dynamic intraventricular gradients. Therefore, pressors with β-agonist properties are generally avoided. In individuals with LVOTO or left ventricular ballooning, these agents should be avoided, as they can exacerbate systolic anterior motion (SAM) of the mitral valve and increase intraventricular gradients by pushing the mitral valve further into the LVOT [78]. In contrast, beta-blockers must be used in such patients to reduce heart rate and contractility, yet vasoconstrictors may be needed to stabilize blood pressure.

Phenylephrine, a pure alpha-adrenergic vasoconstrictor, is widely used to increase systemic vascular resistance (SVR) without inducing inotropic effects. By stimulating α-1 receptors in vascular smooth muscle, it causes vasoconstriction and is well-suited for managing hypotension caused by peripheral vasodilation [79]. Phenylephrine is also preferred in HCM-related cardiogenic shock due to the reflex bradycardia it induces, which can improve cardiac output (CO) by enhancing diastolic filling time, thereby benefiting patients with elevated heart rates and impaired myocardial perfusion. Noradrenaline (norepinephrine) is a potent vasopressor that primarily stimulates α-adrenergic receptors, with mild β-1 adrenergic activity. This combination can increase both contractility and heart rate, which may be harmful in patients with HCM due to the risk of worsening LVOTO. The β-1 adrenergic stimulation increases myocardial contractility, further exacerbating the obstruction. For this reason, beta-blockers, which have the opposite effect by reducing heart rate and contractility, remain the first-line treatment for managing contractility in these patients. Consequently, noradrenaline is less preferred in comparison to other vasoconstrictors, such as phenylephrine, which lacks β-1 activity and therefore avoids aggravating LVOTO. Dopamine’s effects are dose-dependent, requiring high doses (>10 mcg/kg/min) to activate α-1 adrenergic receptors along with D1 and β-1 receptors, leading to increased contractility. However, its role in HCM management remains understudied, and more data are needed.

In a study comparing dopamine and phenylephrine in cats with HCM, dopamine led to significant increases in cardiac index and tissue oxygen delivery, while phenylephrine resulted in a higher systemic vascular resistance index. Both drugs raised systemic and pulmonary blood pressures, but only dopamine was associated with improved cardiac output [80]. This suggests that dopamine may enhance hemodynamic performance more effectively in certain settings, but phenylephrine remains the preferred option in clinical practice for supporting blood pressure without increasing contractility, especially in patients with LVOTO [50,78].

Arginine vasopressin, commonly referred to as vasopressin, may play a significant role in managing hemodynamics in patients with HCM by potentially decreasing pulmonary vascular resistance (PVR) [81]. Research in animal models has shown that vasopressin can induce selective pulmonary vasodilation through the release of nitric oxide, resulting in decreased PVR [82]. This decrease enhances pulmonary blood flow and increases venous return, subsequently elevating preload—a crucial factor in HCM management. Clinical observations support this effect; for instance, the use of vasopressin in neonates with HCM has demonstrated improvements in systemic hypotension and overall hemodynamic stabilization [83]. Furthermore, a study by Balik et al. highlighted the efficacy of vasopressin in patients experiencing LVOTO due to septic shock, showcasing significant results where vasopressin allowed for a reduction in norepinephrine dosage, leading to a decrease in LVOTO and marked hemodynamic improvement [84]. These findings suggest that vasopressin could be a valuable therapeutic option for patients with LVOTO and HCM.

### 4.2. Circulatory Support Devices

Circulatory support devices have become a cornerstone in the rehabilitation of cardiogenic shock caused by acute coronary syndromes or heart failure. However, data on their use in patients with HCM and LVOTO are limited. The unique pathophysiology of HCM presents significant challenges for managing cardiogenic shock and the application of mechanical circulatory support devices (MCSDs). For instance, the smaller left ventricular cavity and thicker walls in HCM have been associated with increased mortality following left ventricular assist device (LVAD) implantation [85]. Reports indicate that HCM is linked to a higher odds ratio of mortality in patients undergoing LVAD implantation (OR 3.8, 95% CI 1.1–12.6, *p* = 0.03), a trend that remains significant even after multivariate adjustment (OR 3.4, 95% CI 1.03–11.2, *p* = 0.04) [85]. Similarly, HCM contributes to a greater likelihood of mortality in patients receiving MCSD (in comparison with patients receiving MCSD without HCM), as evidenced by both univariate (OR 2.5, 95% CI 1.8–3.46, *p* < 0.001) and multivariate logistic regression analyses (OR 2.5, 95% CI 1.8–3.6, *p* < 0.001) [85]. This increased mortality risk persists even after accounting for various demographic, comorbidity, and healthcare setting characteristics, indicating that the pathophysiology of HCM may render patients less optimal candidates for advanced therapies such as intra-aortic balloon pumps (IABPs), Impella devices, and extracorporeal membrane oxygenation (ECMO) [85].

**Figure 2:**
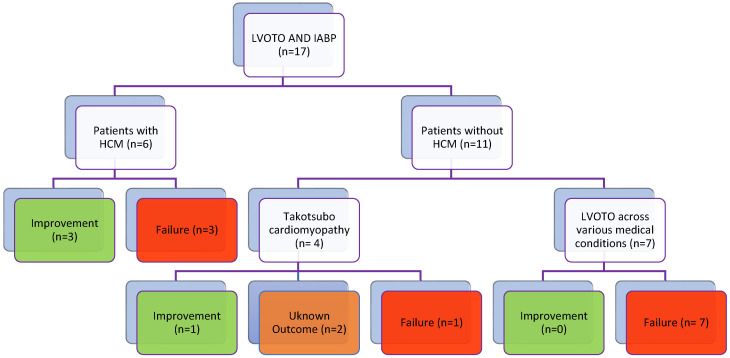
Based on the data of the table outcomes of intra-aortic balloon pump (IABP) in patients with left ventricular outflow tract obstruction (LVOTO), this flow chart presents the outcomes of patients treated with intra-aortic balloon pumps (IABPs) in the context of left ventricular outflow tract obstruction (LVOTO), differentiating between those with hypertrophic cardiomyopathy (HCM) and those without. Out of 17 patients, 6 had HCM, with 3 showing improvement and 3 experiencing treatment failure. Among the 11 patients without HCM, 4 had Takotsubo cardiomyopathy, where 3 improved and 1 failed treatment. The remaining 7 patients had LVOTO due to various medical conditions, with all experiencing failure. This highlights the variable efficacy of IABP based on the underlying condition. **Abbreviations**: IABP—intra-aortic balloon pump, LVOTO—left ventricular outflow tract obstruction, HCM—hypertrophic cardiomyopathy.

Intra-aortic balloon pump (IABP) therapy, while designed to improve coronary perfusion during diastole, can paradoxically worsen or induce new LV outflow obstruction due to presystolic deflation that decreases afterload [50]. This effect is particularly concerning in patients with LVOTO, making IABP contraindicated in such cases [78,86]. Although IABP has been used to address cardiogenic shock resulting from Takotsubo syndrome, the recent IABP-SHOCK II trial’s neutral results and its potential to exacerbate dynamic LVOTO [87] have led to recommendations against its use in these patients. Instead, for those with primary Takotsubo syndrome experiencing deterioration, LVOTO, and low cardiac output, seeking specialist advice regarding ECMO or LVAD as a bridge to recovery is suggested, especially given the favorable prospects for ventricular function recovery [86] (Table 3) (Figure 2).

**Table 3:** The table provides a summary of various studies examining the impact of IABP therapy on patients with HCM and LVOTO, LVOTO due to other medical conditions, and other related conditions. Overall, the outcomes suggest that while IABP may provide benefits in certain cases, it can also lead to adverse effects such as worsening LVOTO, and its management often requires careful consideration of individual patient circumstances. Cases with LVOTO that do not involve HCM are also included due to the limited number of cases specifically featuring HCM and LVOTO. The similar pathophysiology of these two conditions (Takotsubo and MI with LVOTO) enhances our understanding of the IABP therapy.

Author	Setting of IABP	Key Details
Griffin M. et al. [87], 2023	Takotsubo and HOCM	IABP exacerbated LVOTO, causing severe obstruction and worsening shock, and the removal improved hemodynamics.
Noori MAM. et al. [88], 2022	Acute inferolateral myocardial infarction	IABP worsened LVOTO, and reduction in counterpulsation and addition of fluids improved the condition. IABP was reduced in 1:3
O’Brien J. et al. [89], 2021	Takotsubo and LVOTO	IABP rapidly improved hemodynamics and LVOT obstruction, and the patient fully recovered within 6 days.
Sherrid MV. et al. [50], 2021	HCM and LVOTO	Fourteen patients with HCM. IABP counterpulsation was used in 4 and may have aided recovery in 2, but failed to improve shock in the other 2
Bui QM. et al. [90], 2021	Takotsubo cardiomyopathy and LVOTO	IABP worsened hypotension, while its discontinuation improved the condition.
De Backer O. et al. [91], 2014	Takotsubo cardiomyopathy and LVOTO	IABP was used in two patients, with unknown outcomes, and there was no mortality in the LVOTO group.
Takamura T. et al. [92], 2012	Cardiogenic shock and acute myocardial infarction due to stent thrombosis	IABP-induced LVOTO; removal led to complete recovery, and CI was dramatically increased from 1.3 to 2.2 L/min/m^2^.
Hsu PC. et al. [93], 2011	HCM and LVOT obstruction	IABP improved hemodynamics, and LVOT obstruction and pulmonary edema were resolved.
Cohen R. et al. [94], 2006	Acute anterior myocardial infarction	IABP led to dynamic LVOTO, and the removal of IABP resolved obstruction.
Coddens J. et al. [95], 2002	Anterior myocardial infarction complicated with ventricular septal defect—post-VSD repair LVOTO	IABP-induced SAM and mitral regurgitation improved when reduced to 2:1 mode.
Di Chiara A. et al. [96], 2001	Acute myocardial infarction	IABP and inotropes worsened LVOTO, and recovery followed their discontinuation.
Tse RW. et al. [97], 1996	Acute anterior myocardial infarction	IABP placement led to deterioration, and a dynamic LVOT gradient was detected.
Nakhjavan FK. et al. [98], 1983	Post-aortocoronary bypass graft LVOT obstruction	IABP and dobutamine-induced LVOTO after CABG, which resolved with volume load.

**Abbreviations:** IABP—intra-aortic balloon pump; HCM—hypertrophic cardiomyopathy; LVOTO—left ventricular outflow tract obstruction; CI—cardiac index; SAM—systolic anterior motion.

The Impella catheter offers partial left ventricular support by delivering blood into the proximal aorta and reducing preload. However, in HCM patients, this can decrease left ventricular cavity size, which may induce SAM of the mitral valve and further increase LVOTO [78]. A similar phenomenon may occur with the TandemHeart device. Despite these challenges, short-term mechanical circulatory support, such as the Impella, may be considered a bridge to recovery in patients with refractory cardiogenic shock and LVOTO [78]. In one notable case, the timely placement of an Impella CP assist device in a patient with Takotsubo syndrome and severe mitral regurgitation led to improved hemodynamics and resolution of LVOTO, allowing for successful recovery and discharge after five days of mechanical support [99]. However, specifically for patients with HCM and LVOTO, only two documented cases exist, in which one patient was successfully weaned from Impella, while the other required ECMO to survive cardiogenic shock [50,100].

Conversely, venoarterial extracorporeal membrane oxygenation (VA-ECMO) provides full circulatory support and can rapidly augment cardiac output and coronary perfusion [50]. There are only a few cases of HCM with cardiogenic shock that responded to treatment with ECMO and were successfully weaned from it (Table 4). It tends to respond favorably in patients with HCM, particularly when afterload is mildly increased [50]. One case reported the successful management of a patient with HCM in shock due to Takotsubo cardiomyopathy, requiring VA-ECMO, which showcased its potential as a viable option. After ECMO removal, the patient experienced hemodynamic compromise due to atrial fibrillation, leading to the decision for surgical myectomy [101]. However, in some cases, patients with HCM may not be successfully weaned from ECMO, necessitating further interventions, such as septal myectomy or other procedures to reduce septal myocardium. Failure to wean from ECMO is not attributed to the incompatibility of ECMO in HCM patients but rather to the critical condition of the patient at the time. ECMO is generally well-tolerated in these patients [102,103] (Table 4).

In summary, the use of IABP and Impella in patients with HCM, apical ballooning, or obstruction is associated with an increased risk of LV outflow obstruction, while ECMO may improve hemodynamics and is often the preferred choice [50]. The findings by Hussain et al., indicating heightened mortality rates in HCM patients undergoing MCSD, may stem from the aggregation of all devices (IABP, Impella, and ECMO) into a single category; a separation of these groups could potentially reveal more favorable outcomes with ECMO [85]. Thus, clinical trials are essential for elucidating the role of these devices in this complex patient population.

**Table 4:** This table summarizes various cases involving the use of mechanical circulatory support devices, specifically Impella and ECMO, in patients with different medical conditions, including HCM, Takotsubo cardiomyopathy, and LVOTO, due to other medical conditions. The outcomes highlight the effectiveness of these devices in managing cardiogenic shock and improving hemodynamic stability, with many patients successfully weaned from support. Both HCM and non-HCM cases are presented to provide insights into the therapeutic potential and challenges of using these devices in managing LVOTO.

Data on Patients with HCM			
**Cases—Author**	**Medical Condition**	**Device Used (IMPELLA/ECMO)**	**Outcome**
Mishra SK. et al. [100], 2021	Takotsubo stress cardiomyopathy with LVOTO and **HCM**	Impella CP	**Success weaning**, Impella removed after 72 h, hemodynamically improved
Sherrid MV. et al. [50], 2021	14 patients with **HOCM**	Impella 1 patient ECMO 3 patients	Impella patient: **Required ECMO** after Impella ECMO patients: **Success with VA-ECMO** (4–13 days on ECMO); all patients survived their acute shock events
Caniato F. et al. [101], 2021	**HOCM**	ECMO	**Successful weaning** from VA-ECMO. 6 h later, hemodynamic compromise due to atrial fibrillation and surgical myectomy was decided
Husaini M. et al. [103], 2020	**HCM** and LVOTO due to Takotsubo	ECMO	**Weaning failure**. Surgical myectomy required and VA-ECMO weaning succession after surgery
Kobayashi T. et al. [104], 2020	**HOCM**	ECMO	Successful recovery after percutaneous septal myocardial ablation under ECMO support
Sossalla S. et al. [102], 2019	**HOCM**	ECMO	**ECMO weaning failed** Bailout redo transcoronary ablation of septal hypertrophy (TASH) performed, ECMO weaned, LVOT gradient significantly reduced
**Data on patients with LVOTO, without HCM**			
Benak A. et al. [105], 2022	Takotsubo cardiomyopathy with LVOTO and severe MR	Impella CP	**Success of weaning** Recurrence of LVOTO and MR after Impella removal with mild pulmonary congestion
Attisano T et al. [99], 2020	Takotsubo syndrome with LVOTO and MR	Impella CP	**Success weaning**, device removed after 5 days and LVOTO and MR resolved and discharged
Mohammedzein A. et al. [106], 2019	Takotsubo cardiomyopathy with LVOTO	Impella 2.5	**Success weaning**, Impella removed on the 8th day, hemodynamic status improved
Beneduce A. et al. [107], 2019	Takotsubo syndrome with LVOTO and severe MR	Impella 2.5	**Success weaning**, Impella removed after 72 h, LV function and perfusion improved
Oxlund C.S. et al. [108], 2018	Post cardiac arrest due to acute coronary syndrome and LVOTO with MR	ECMO	**Success weaning**, immediate improvement in hemodynamics, and resolved SAM after ECMO placement, removed day 4
Bonacchi M. et al. [109], 2009	Takotsubo cardiomyopathy with LVOTO	ECMO	After recovery of LV function reduction in MR (grade) and LVOT obstruction (day 5), ECMO successfully removed

**Abbreviations:** Impella—IMPELLA; extracorporeal membrane oxygenation—ECMO; hypertrophic cardiomyopathy—HCM; left ventricular outflow tract obstruction—LVOTO; mitral regurgitation—MR.

### 4.3. Left Ventricle Assist Devices (LVAD)

Left ventricular assist devices (LVADs) are not typically used as an emergency measure in cardiogenic shock. However, patients with HCM who experience shock are at high risk and may require heart transplantation. In such cases, an LVAD may be considered a bridge to more definitive treatment. Although LVADs are not typically recommended for these patients due to the expectation of reversing systolic dysfunction once the outflow obstruction is resolved, there is growing recognition of their successful use in select cases. The European Society of Cardiology (ESC) mentions LVADs as a possible treatment for HCM patients with advanced heart failure, although the evidence level is classified as C [110]. In addition, these devices are not used as an emergency intervention in cardiogenic shock but are reviewed in this text due to the importance of LVADs and supportive measures for patients who may experience cardiogenic shock from hypertrophic cardiomyopathy (HCM). The important measures taken after stabilizing these patients in shock serve as a bridge to a definitive solution. In patients with HCM, durable LVADs have been successfully used as a bridge to transplantation, although anatomical challenges like small ventricular chamber sizes and muscle bundles may obstruct the inflow cannula [50]. Despite these concerns, patients with HCM undergoing continuous-flow (CF) LVAD implantation show promising results. In a cohort of 104 HCM patients who underwent CF LVAD implantation, their survival and adverse event rates were comparable to those of patients with dilated cardiomyopathy (DCM) [111]. However, for those with very small ventricles, survival outcomes were worse.

Comparing outcomes between HCM/restricted cardiomyopathy (RCM) patients and traditional DCM patients revealed no significant difference in early mortality (12.5% vs. 9.3%, *p* = 0.57) or length of hospital stay (11 days vs. 18.5 days, *p* = 0.51). However, HCM/RCM patients had higher right atrial pressure (18 mm Hg vs. 12 mm Hg, *p* = 0.03) and lower pump flow rates after LVAD implantation (4.3 L/min vs. 5.2 L/min, *p* = 0.001) [112]. Despite these challenges, mortality rates in patients treated with LVADs due to HCM-related heart failure were similar to those with dilated or ischemic cardiomyopathy.

Nevertheless, a large US study demonstrated that underlying HCM is associated with a significantly increased risk of mortality following LVAD implantation, with an odds ratio (OR) of 3.8 (95% CI 1.1–12.6, *p* = 0.03), and this risk remained after multivariate adjustment (OR 3.4, 95% CI 1.03–11.2, *p* = 0.04) [85]. This underscores the importance of patient selection and individualized care when considering LVADs for HCM patients.

### 4.4. Invasive Treatment

An invasive treatment option for HCM patients with LVOTO is recommended when medications fail. However, there is no evidence supporting the use of invasive procedures in asymptomatic patients, regardless of the severity of their LVOTO. Some retrospective studies indicate that individuals with high LVOT gradients, even if minimally symptomatic, have a higher mortality rate compared to those without elevated gradients [16].

Early intervention may result in fewer complications and better long-term outcomes [113]. Invasive treatment to reduce LVOTO should be considered for patients with an LVOT gradient of ≥50 mmHg, severe symptoms (NYHA functional class III–IV), and/or exertional or unexplained recurrent syncope, despite the use of maximally tolerated medical therapy [16]. Of course, patients who experienced cardiogenic shock due to HCM are candidates for invasive treatment.

There are two main invasive options available, namely, surgical ventricular septal myectomy or alcohol septal ablation (ASA). In experienced centers, ASA, which involves injecting alcohol into a septal perforator artery to create a localized septal scar, has shown similar outcomes to surgery in terms of gradient reduction [16]. While there are no randomized trials directly comparing surgery and ASA, several meta-analyses have demonstrated that both procedures significantly improve functional status with similar procedural mortality rates [114,115,116].

These data, however, refer to patients in a stable condition, not those in cardiogenic shock. In certain cases, patients in cardiogenic shock may require invasive treatments, such as septal reduction, to successfully wean off mechanical circulatory support devices. Some patients have been successfully treated with both alcohol septal ablation [102,104,117] and surgical myectomy [118] while in cardiogenic shock. In these instances, patients may first be stabilized with circulatory support devices before undergoing invasive procedures. Sherid et al. recently reported successful outcomes for patients in cardiogenic shock who were stabilized with either medical treatment or devices before undergoing ASA or surgery. They suggested that if tapering from VA-ECMO is not possible due to recurrent obstruction, septal reduction should be considered the next step [50].

**Figure 3:**
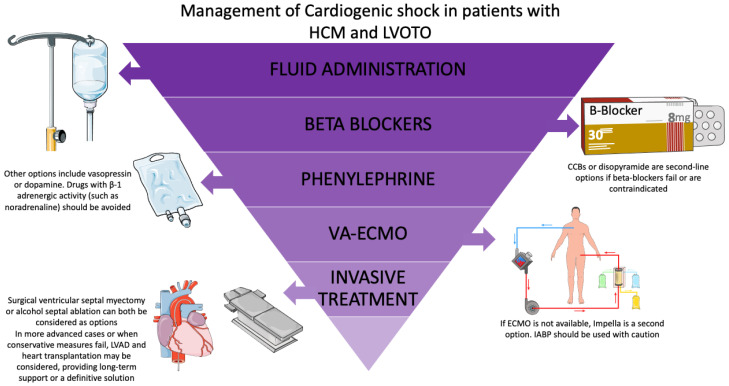
Suggested protocol for the management of cardiogenic shock due to LVOTO in HCM patients. Abbreviations: calcium channel blockers (CCBs), extracorporeal membrane oxygenation (ECMO), hypertrophic cardiomyopathy (HCM), left ventricular assist devices (LVADs), left ventricular outflow tract obstruction (LVOTO). Parts of the figure were drawn by using pictures from Servier Medical Art. Servier Medical Art by Servier is licensed under a Creative Commons Attribution 4.0 Unported License (https://creativecommons.org/licenses/by/4.0/, accessed on 9 October 2024).

Cardiogenic shock in HCM primarily arises from ischemia and myocardial fibrosis. The underlying pathogenesis is complex and not entirely understood, as ischemia can occur not only within the hypertrophied septum but also in the free wall of the left ventricle [36,37,38]. This phenomenon can lead to the development of “end-stage” HCM, characterized by a dilated cardiomyopathy phenotype and severe systolic dysfunction. In such cases, cardiogenic shock may ensue, necessitating advanced therapeutic interventions. For patients who progress to end-stage HCM, cardiac transplantation may become the only viable option and should be considered in the management plan [9,16]. Until transplantation, patients may be placed in a bridging state, supported by mechanical circulatory devices such as ECMO or LVAD, to stabilize hemodynamics and await definitive treatment (Figure 3) [110,111].

## 5. Key Points

- Cardiogenic shock in HCM patients is a complex condition and may present as classical heart failure shock but more commonly as shock due to LVOT obstruction.
- Cardiogenic shock resulting from LVOT obstruction has a different pathophysiology and consequently requires different management strategies.
- Intravenous fluids and beta-blockers are the first-line options.
- Circulatory support devices are used infrequently in these patients, and data on their effectiveness are limited; however, their use is extremely promising, particularly the application of VA-ECMO.
- In patients with HCM and cardiogenic shock, devices such as Impella and IABP should generally be avoided, particularly IABP, as they can exacerbate LVOTO and worsen hemodynamics. ECMO may be a better exit strategy in severe cases, offering support while avoiding the complications associated with these devices.
- In situations where pharmaceutical treatment and circulatory support devices fail, invasive treatments such as surgical ventricular septal myectomy or alcohol septal ablation may be employed in an emergency setting.

## 6. Conclusions

Patients with HCM often have an excellent overall prognosis, and advanced heart failure or cardiogenic shock is uncommon. However, managing cardiogenic shock in HCM patients presents unique challenges due to the distinct pathophysiology, particularly left ventricular outflow tract (LVOT) obstruction, which differentiates it from other types of cardiogenic shock. Initial management typically involves intravenous fluids and beta-blockers. Circulatory support devices, especially ECMO, have been used successfully in some cases, though their use remains infrequent. In emergencies, invasive options like surgical ventricular septal myectomy or alcohol septal ablation may be necessary. However, there are limited data on the optimal timing and choice of these interventions. Given the lack of definitive data, clinicians must rely on their judgment based on local availability and expertise, highlighting the need for more clinical trials in these rare but severe scenarios.

## Figures and Tables

**Table 1:** Explanation of FEW, BEW, FCW, and BCW.

In a healthy heart, coronary blood flow is primarily influenced by two key waveforms: the forward compression wave (FCW) created by ventricular contraction, which propels blood into the coronary arteries, and the backward expansion wave (BEW), arising from the release of pressure in the microcirculation during ventricular relaxation. As ventricular contraction begins to wane, the FCW decelerates coronary flow. Additionally, during systole, early and late backward compression waves (BCWs) manifest as the intramyocardial vessels experience compression [39]. BEWs, generated when the ventricle relaxes, lead to a reduction in ventricular pressure and a “suction” effect that can impede forward coronary flow during early diastole. This results in the deceleration of coronary flow rather than acceleration. On the other hand, forward expansion waves (FEW) contribute to coronary flow acceleration. FEWs occur when blood is ejected from the left ventricle, propelling coronary blood forward, and they are associated with the contraction phase, playing a crucial role in increasing coronary flow during systole and the transition into early diastole.

## Data Availability

Not applicable.
